# Human CD4-Binding Site Antibody Elicited by Polyvalent DNA Prime-Protein Boost Vaccine Neutralizes Cross-Clade Tier-2-HIV Strains

**DOI:** 10.21203/rs.3.rs-3360161/v1

**Published:** 2023-10-20

**Authors:** Shixia Wang, Kun-Wei Chan, Danlan Wei, Xiuwen Ma, Shuying Liu, Guangnan Hu, Saeyoung Park, Ruimin Pan, Ying Gu, Alexandra F. Nazzari, Adam S. Olia, Kai Xu, Bob C. Lin, Mark K. Louder, Nicole A. Doria-Rose, David Montefiori, Michael S. Seaman, Tongqing Zhou, Peter D. Kwong, James Arthos, Xiang-Peng Kong, Shan Lu

**Affiliations:** 1 Department of Medicine, University of Massachusetts Chan Medical School, Worcester, MA 01655, USA; 2 Department of Biochemistry and Molecular Pharmacology, New York University Grossman School of Medicine, New York, NY 10016, USA; 3 Laboratory of Immune Regulation, National Institute of Allergy and Infectious Diseases, NIH, Bethesda, MD 20892, USA; 4 SYL Consulting, Thousand Oak, CA 91320, USA; 5 Vaccine Research Center, National Institute of Allergy and Infectious Diseases, NIH, Bethesda, MD 20892, USA; 6 Department of Surgery, Duke University, Durham, NC 27710, USA; 7 Center for Virology and Vaccine Research, Beth Israel Deaconess Medical Center, Harvard Medical School, Boston, MA 02115, USA

**Keywords:** CD4-binding site, DNA immunization, envelope glycoprotein, gp120, HIV-1, monoclonal antibody, Tier-2 neutralization, vaccine

## Abstract

The vaccine elicitation of HIV-neutralizing antibodies with tier-2-neutralization breadth has been a challenge. Here, we report the isolation and characteristics of a CD4-binding site specific monoclonal antibody, HmAb64, from a human volunteer immunized with a polyvalent gp120 DNA prime-protein boost vaccine. HmAb64 derived from heavy chain variable germline gene IGHV1-18, light chain germline gene IGKV1-39, and had a 3^rd^ heavy chain complementarity determining region (CDR H3) of 15 amino acids. On a cross-clade panel of 208 HIV-1 pseudo-virus strains, HmAb64 neutralized 21 (10%), including tier-2 neutralization resistant strains from clades B, BC, C, and G. The cryo-EM structure of the antigen-binding fragment of HmAb64 bound to a conformation between prefusion closed and occluded open forms of envelope trimer, using both heavy and light CDR3s to recognize the CD4-binding loop, a critical component of the CD4-binding site. A gp120 subunit-based vaccine can thus elicit an antibody capable of tier 2-HIV neutralization.

## Introduction

While antiviral treatment has successfully halted disease progression in many people infected with HIV-1 ^1^, and various innovative preventive measures have slowed the global AIDS pandemic ^2^, an estimated 1·5 million new HIV infections occurred in 2021 worldwide ^3^. Therefore, the development of a safe and effective HIV vaccine remains an important objective toward ending the HIV epidemic, as such a vaccine offers low-cost, long-lasting protection to the global population, especially when other approaches are either expensive or have various side effects.

One of the bottlenecks to an effective HIV-1 vaccine is the difficulty in raising protective antibody responses, capable of neutralizing the diverse tier-2 neutralization resistant viruses that typify HIV-1 transmission. Broadly neutralizing antibodies capable of such neutralization have been a major focus of HIV-1 vaccine research for the past several decades, based on their broad cross-clade neutralization shown by *in vitro* assays ^4–7^ as well as their ability to protect monkeys *in vivo* from infection by simian-human chimeric virus challenge. A major challenge for the HIV vaccine field is the inability to elicit potent broadly neutralizing responses by vaccination, either in standard vaccine-test animals or human vaccine trials. At the same time, remarkable progress has been made in isolating human monoclonal antibodies (mAbs) from HIV infected patients with high breadth and potency, as well as structural definition of the recognition of these antibodies on the HIV-1 envelope (Env) spike ^6,8–11^.

The HIV-1 Env spike on the surface of HIV-1 virions comprises trimers of heterodimers formed by the two Env subunits, gp120 and gp41, which are the targets of neutralizing antibodies. The infection process of HIV-1 starts when gp120 binds to the receptor CD4 and co-receptors CCR5 or CXCR4 on host cells. Conformational changes induced by Env interaction with receptors triggers membrane fusion between virus and host cell.

Broadly neutralizing antibodies can be categorized into several major groups based on their target sites: (1) the CD4-binding site (CD4bs), (2) the V1V2 apex, (3) the V3-glycan, (4) the fusion peptide (FP), (5) the gp120-gp41 interface, and (6) the membrane-proximal external region (MPER) ^12^. Among these sites, the CD4bs on HIV-1 gp120 is significant because it mediates the initial step of virus-host interaction. It is functionally conserved for interaction with the CD4 receptor and is a site of vulnerability that can be targeted by antibodies to neutralize HIV-1 ^9,13^.

Even though the CD4bs on gp120 is recessed and optimized to bind the single-headed Ig-like CD4 ^14^, the human immune system nonetheless targets this site with CD4bs antibodies. Several classes of antibodies that effectively neutralize HIV-1 through CD4 mimicry have been described including those derived from VH1-2 germline such as the well-characterized VRC01 class of antibodies ^4,6,11,15–17^, those derived from the closely related VH1-46, such as 8ANC131 ^6,10^, and others like IOMA and similar antibodies ^18^. Additional CD4-binding site antibodies include those that utilize their heavy chain third complementarity determining regions (CDR H3s) to recognize the CD4-binding site; these types of antibodies have been isolated from multiple donors, each with a germline origin that is different from VH1-2 or VH1-46 ^17,19^. It is known that the breadth of cross-reactive neutralizing activity such as anti-CD4bs antibodies arise in some patients only after 2 to 4 years of continued HIV-1 infection ^20,21^, imposing a major challenge to HIV vaccines which are delivered to humans in a few injections.

Here we report the isolation and characterization of a CD4bs-specific mAb from a human volunteer who received a polyvalent gp120 DNA prime-protein boost HIV vaccine in a previously reported phase I study DP6-001 ^22,23^. This antibody derived from a germline different from VH1-2 or VH1-46 and neutralized several tier-2 resistant HIV-1 strains from different clades, albeit with moderate potency.

## Results

### Immunization of human volunteers and isolation of a novel CD4bs mAb

**Figure 1 |:**
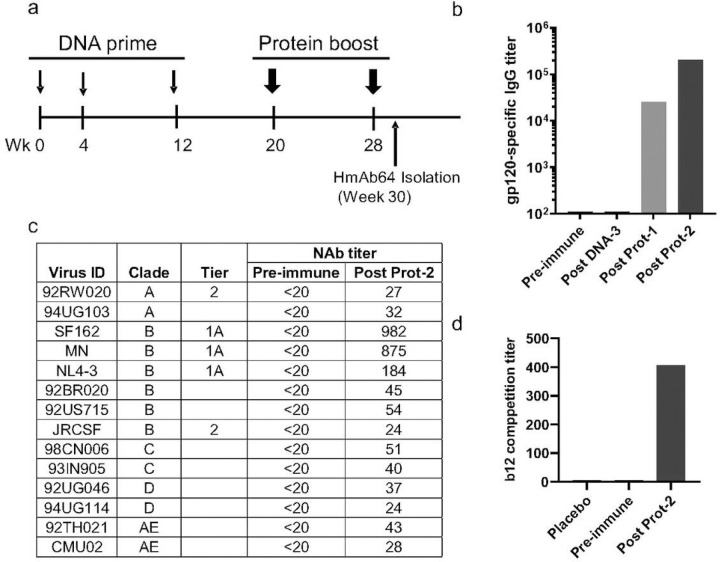
DNA prime and protein boost elicited serum HIV-1 Env specific neutralizing antibody responses targeting the CD4-binding site. (a) Immunization scheme. Serum samples were collected two weeks after each immunization and PBMCs were collected two weeks after the last immunization. (b) Development of gp120-specific IgG titers following key immunization steps. (c) Neutralization with the volunteer serum at peak level against a panel of pseudotyped viruses expressing primary Env antigens from different subtypes of HIV-1 as indicated. (d) Competition of volunteer sera against CD4bs-specific mAb b12-captured infection to target cells by HIV-1 JR-FL Env-expressing pseudovirus as previously reported ^23^. The ability of serum competition is presented as the serum dilutions that can achieve 50% inhibition. Unrelated human serum was used as the placebo control.

In our previously reported Phase I clinical trial (DP6-001) of a polyvalent gp120 DNA prime-protein boost HIV vaccine PDPHV, healthy adult volunteers who were negative for HIV infection first received three immunizations of a DNA plasmid mixture including five expressing gp120 antigens from diverse HIV-1 subtypes, followed by two protein immunizations of five recombinant gp120 proteins matching those in the DNA prime immunization (Fig. 1a). Peripheral blood collected at two weeks after the second protein immunization showed high level of anti-gp120 IgG titers and cross clade neutralizing antibody responses in volunteers’ immune sera ^22^. From DP6-001 study volunteers, a panel of gp120-specific human monoclonal antibodies were isolated including those showing potent and cross clade ADCC activities as previously reported ^24^. In the current study, PBMC collected from one DP6-001 volunteer (ABL009) were used to identify mAbs with potential neutralizing antibody activities. ABL009 serum had increasing titers of gp120-specific IgG following the prime-boost immunization (Fig. 1b), and more importantly ABL009 serum showed low titer but broad neutralizing antibody activities against not only the sensitive Tier 1A virus but also less sensitive viruses from subtypes A, B, C, D and E conducted by the Monogram assay (Fig. 1c). A cell-based competition assay further indicated the presence of CD4bs activities based on the ability of ABL009 serum competing against a well-known CD4bs mAb b12 (Fig. 1d).

**Figure 2 |:**
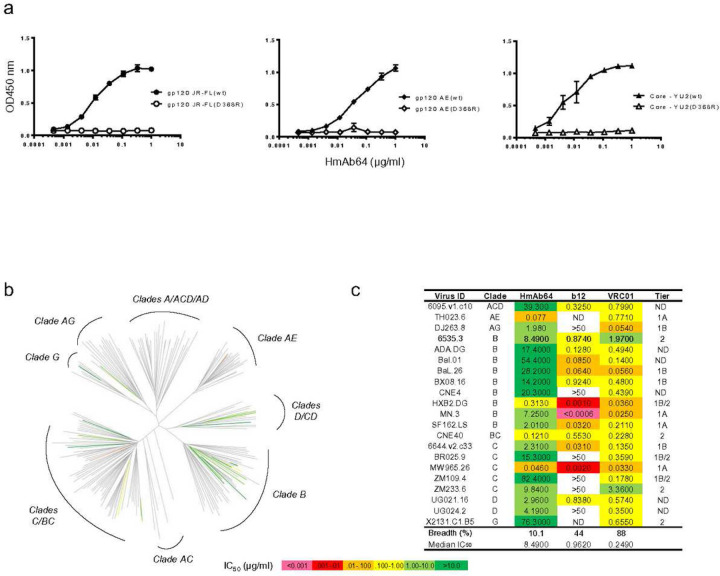
Vaccine elicited antibody HmAb64 targets the CD4-binding site and neutralizes tier-2 HIV-1 strains. (a) HmAb64 binding to gp120 Env proteins. HmAb64 showed strong binding to wild type gp120 antigens from multiple HIV-1 isolates but not to their D368R mutants, indicating CD4-binding site specificity. (b) Neutralization analysis. HmAb64 neutralized 21 viruses (10%) from diverse clades of the 208-strain NIAID panel. (c) Neutralization titers and tier designation of VRC panel viruses neutralized by HmAb64.

One mAb (HmAb64) was isolated from this volunteer which showed strong binding to the wild type gp120 Env proteins from multiple HIV-1 isolates (JR-FL, AE, YU2) but not to their D368R mutant counterparts (Fig. 2A). Because CD4bs is lost in D368R mutants ^14,25,26^, our result suggested that HmAb64 targets at CD4bs of these gp120 proteins.

Defined by IMGT, VH and VL genes of HmAb64 were identified and assigned to germline IGHV1-18*01 and IGKV1-39*01. The CDRH3 length was 15 aa and CDRL3 length was 9 aa according to Kabat definition. The overall mutation frequency from the germline for VH and VL was at 4.1% and 4.2%, respectively.

### The breadth of HmAb64’s neutralizing activities

By using the standard TZM-bl assay, HmAb64 was shown to neutralize Tier 1A and Tier 1B HIV-1 primary isolates from subtypes B, C, AE, and AG (MS.3, SF163.LS, Bx08.16, 6535.3, MW965.26, CH0505.w4.3, 92BR025.9, DJ263.8 and TH023.6) (Table 1). At the same time, 14 HIV-1 isolates included in the same assay were not neutralized at the IC50 concentration of 50 ug/ml. In a separate assay, HmAb64 was tested against the standard VRC panel of 208 HIV-1 viruses, and it was able to neutralize 21 viruses from diverse subtypes (10% of the panel) (Fig. 2c). Notably, several of the isolates including those from clades B, BC, C, and G were Tier-2 neutralization resistant (Fig. 2d).

### HmAb64 binding activity and CD4bs specificity

**Figure 3 |:**
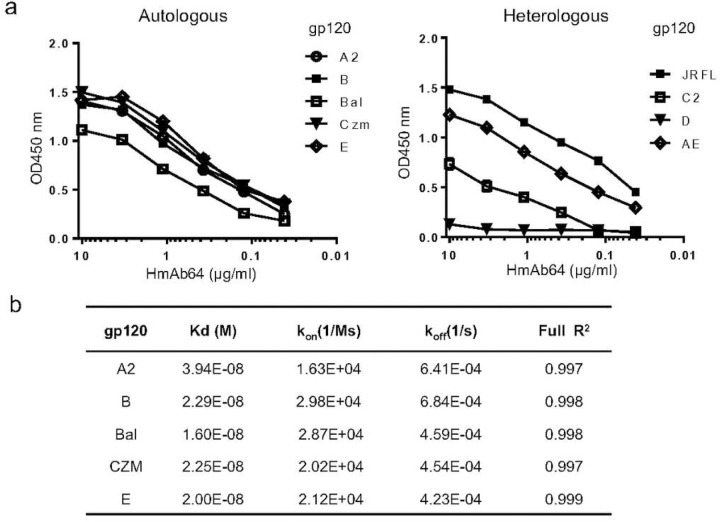
Binding of HmAb64 to autologous or heterologous gp120 antigens. (a) Binding to either autologous gp120 antigens included in the PDPHV vaccine (left) or heterologous gp120 antigens (right). (b) Surface plasma resonance binding kinetics of HmAb64.

HmAb64 showed wide breadth against HIV-1 gp120 antigens from various subtypes. It demonstrated strong affinity to the five autologous gp120 antigens (A2, B, Bal, Czm and AE) used in the original PDPHV vaccine formulation (Fig. 3a, left panel). The binding affinity was observed in the low nanomolar range between 1.6×10^−8^ M to 3.9×10^−8^ M (Fig. 3b). HmAb64 was also able to bind to other unrelated (heterologous) gp120 antigens, though their levels of binding affinities differ (Fig. 3a, right panel).

**Figure 4 |:**
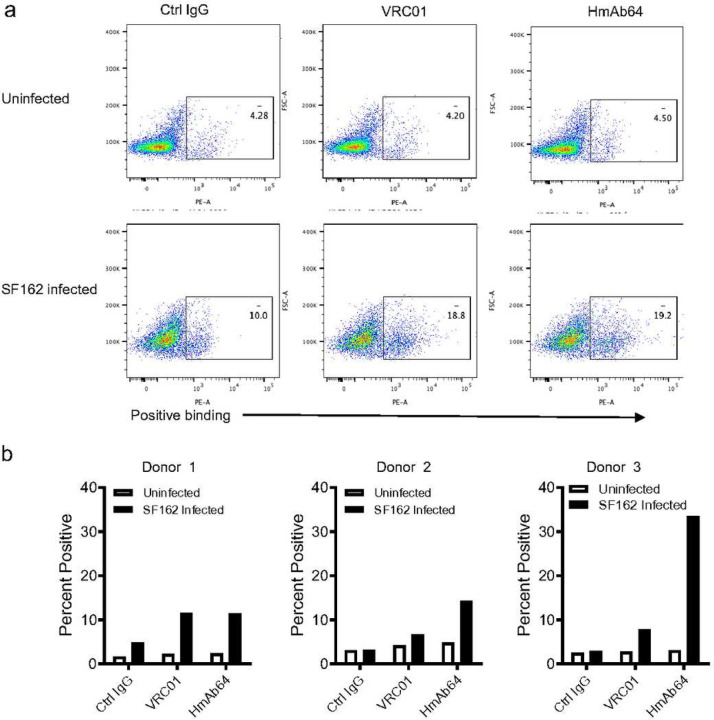
Binding of HmAb64 to HIV-1 on cell surface. (a) Examples of FACS analyses and gating showing the specific binding of HmAb64 to HIV-1 SF162 infected human CD4+ T cells but not to uninfected cells. CD4bs mAb VRC01 was used as the positive control and normal human IgG was used as the negative control. (b) Percentage of HmAb64 binding to SF162 infected CD4+ T cells using cells from three individual human donors.

The binding of HmAb64 to HIV-1 virus was then verified in cells infected with HIV-1. HmAb64 was able to bind HIV-1 isolate SF162 infected CD4+ T cells but not to uninfected cells, similar to the positive control VRC01, a well characterized CD4bs mAb (Fig. 4a). The negative control human IgG did not exhibit such specific binding. This binding specificity was next tested using multiple human donors. HmAb64 was able to have consistent specific binding to SF162 infected CD4+ T cells from different human donors and the binding level is similar or higher than those of VRC01 (Fig. 4b).

**Figure 5 |:**
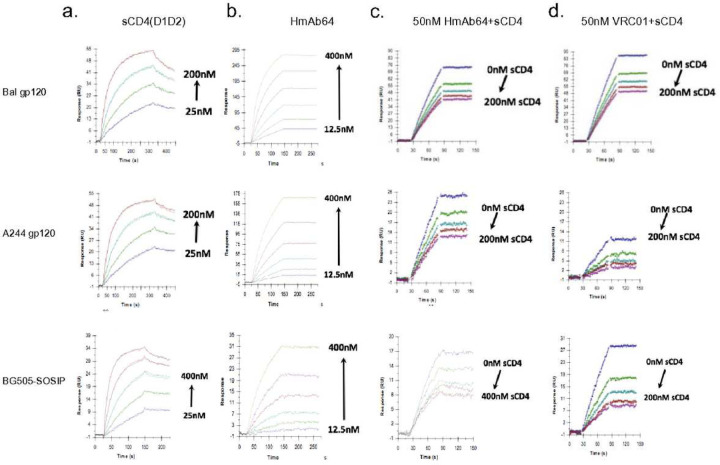
Surface plasmon resonance assay of HmAb64 binding to HIV-1 Env antigens. (a) Titration of sCD4 (D1D2 domain) binding to various HIV-1 Env antigens (Bal gp120, A244 gp120 and BG505-SOSIP). (b) Binding kinetics of HmAb64 to HIV-1 Env antigens. (c) Binding kinetics of HmAb64 to HIV-1 Env antigens in the presence of increasing amount of sCD4(D1D2). (d) Binding kinetics of VRC01 to HIV-1 Env antigens in the presence of increasing amount of sCD4(D1D2).

The CD4bs specificity of HmAb64 to Env proteins from different HIV-1 isolates was further investigated in the presence of soluble CD4 (sCD4) in a Biacore-based assay. HmAb64 was able to bind to gp120 proteins from Bal or A244 viral isolates as well as BG505-SOSIP, and such binding could be blocked by sCD4 in a dose-dependent manner, similar to VRC01. Together, these results confirm that HmAb64 is indeed a CD4bs specific mAb (Fig. 5).

**Figure 6 |:**
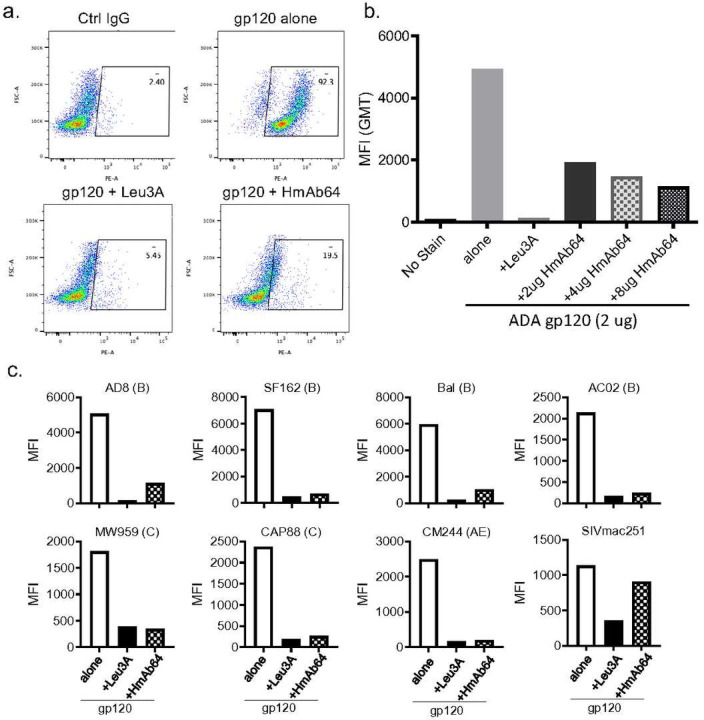
HmAb64 blocked binding of gp120 Env proteins to human CD4+ T cells in a FACS-based assay. (a) HmAb64 blocked binding of gp120 to CD4+ T cells. CD4-binding site antibody Leu3A was used as a positive control. (b) HmAb64 blocking is dose dependent. (c) HmAb64 blocked binding of diverse gp120 antigens to CD4+ T cells. Env protein from SIVmac251 was used as a negative control.

Furthermore, HmAb64 was able to block the binding of gp120 Env proteins from HIV-1 isolates to human CD4+ T cells in a FACS based assay. The blocking by HmAb64 is similar in magnitude to that of Leu3a, a well-known CD4-directed mAb ^27^ (Fig. 6a). The blocking was dose dependent and could be observed with the dose of HmAb64 as low as 2 μg (Fig. 6b). HmAb64 was able to have such blocking effect to gp120 Env proteins from a wide diversity of HIV-1 isolates including SF162, Bal, AC02, AD8, CAP88, MW959 and CM244. The control Env from non-human primate virus SIV251 showed low level binding which could not be blocked by HmAb64 (Fig. 6c), confirming the specificity of HmAb64 to the CD4bs of HIV Env proteins.

HmAb64 was also tested for its ability to bind to autoantigens such as dsDNA, ssDNA, LPS and human insulin (Supplement Fig 1). It showed only minimal binding to these autoantigens, similar to the CD4bs specific human mAb VRC01). In contrast, another human mAb 4E10 which is specific for gp41 of HIV-1 Env had much higher binding to these autoantigens as previously reported ^28^, similar to several agents (ANA NC, ANA PC1, ANA PC2) that are known to bind to autoantigens.

### Structure of HmAb64 in complex with HIV-1 Env

**Figure 7 |:**
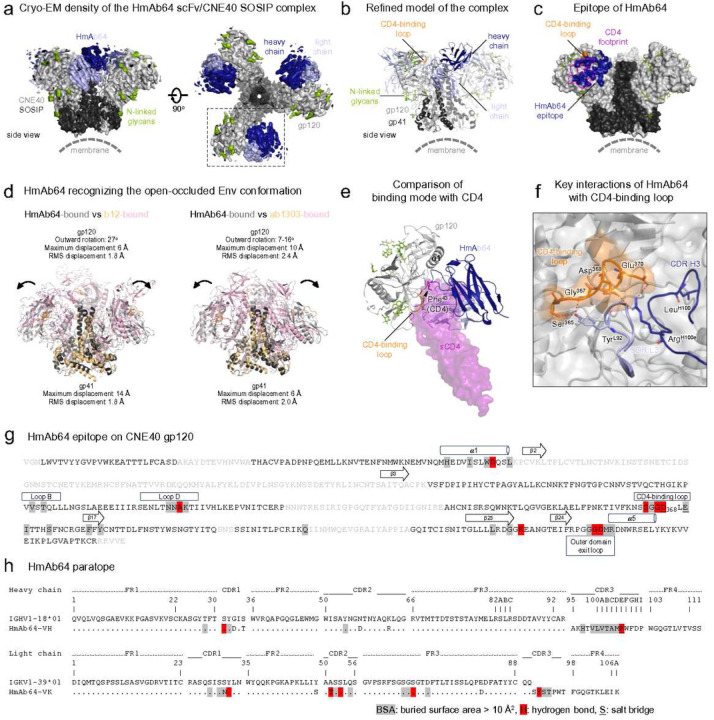
Cryo-EM structure of HmAb64 in complex tier 2 CNE40 SOSIP Env. (a) Cryo-EM density of HmAb64 in complex with CNE40 Env in two perpendicular views. The gp120, gp41, N-linked glycans as well as heavy and light chains of bound HmAb64 were colored light gray, dark gray, green, blue and slate blue, respectively. (b) Refined cryo-EM structure of HmAb64 in complex with CNE40 Env. The CD4-binding loop in gp120 was colored orange. (c) Epitope of HmAb64 in CNE40 Env. It is of note that the epitope (blue) located only to one side of the CD4-binding loop (orange). (d) HmAb64 recognized the open-occluded conformation similar to those recognized by antibodies b12-and ab1303. (e) HmAb64 and CD4 recognized the same side of the CD4-binding loop. (f) Key HmAb64 interactions with the CD4-binding loop of gp120. (g) Mapping of HmAb64 epitope on CNE40 gp120. Epitope residues were highlighted in gray shade with those forming hydrogen bonds colored red and those forming salt bridges underlined. Amino acids that were disordered in the cryo-EM structure were shown in light gray font. Secondary structures of the antibody-binding regions were shown for reference. (h) Mapping of hmAb64 paratope. Paratope residues were highlighted in gray shade with those forming hydrogen bonds colored red and those forming salt bridges underlined. Only two SHM residues were involved in direct contact with gp120. The sequences were numbered according to the Kabat nomenclature.

Structural characterization of HmAb64 was carried out by both protein crystallography and single particle cryogenic electron microscopy (cryo-EM). The antigen-binding fragment (Fab) of HmAb64 was first crystallized, and its structure determined to 2.8 Å resolution (Supplementary Fig. 2 and Supplementary Table 1). The crystal structure showed that HmAb64 in its apo form has a relatively flat antigen binding site, like many known CD4bs bnAbs. We then attempted to obtain a complex structure of HmAb64 Fab with an HIV Env trimer. However, this complex tends to form a mesh-like lattice structure (data not shown), likely induced by the interaction between the constant domain of HmAb64 Fab, which introduced additional complication for the cryo-EM data analysis. We therefore produced a single chain variable fragment (scFv) of HmAb64, and used it to form a complex with an HIV Env trimer from strain CNE40, stabilized by DS-SOSIP along with six arginines at the gp120-gp41 boundary to enhance cleavage ^29–32^. We collected single particle cryo-EM data and obtained a cryo-EM structure of the complex, with 3 HmAb64 scFvs bound at 3.7 Å resolution (Fig. 7a, b and Supplementary Fig. 3). HmAb64 bound to the CD4 supersite ^17^ with both heavy and light chains contacting the CD4-binding loop. Unlike other CD4bs antibodies, the epitope of HmAb64 was confined to a single gp120 protomer, with recognition occurring primarily with the inward side of the CD4-binding loop (Fig. 7c, d).

The cryo-EM structure of HmAb64 scFv in complex with CNE40 SOSIP also revealed that HmAb64 binds the CNE40 trimer in a conformation between prefusion close and occluded open form, similar to that recognized by b12 and other CD4-binding site antibodies ^33,34^, but distinct from the fully open form of the trimer induced by CD4-binding. When superposed on the gp41 HR1 helix, the HmAb64-bound gp41 aligned relatively well with that of ab1303-bound (RMS displacement 2.6 Å and maximum displacement 5 Å), but the gp41 in the b12-bound Env showed higher degree of conformational difference, especially in the HR2 region (RMS displacement 1.8 Å and maximum displacement 14 Å) (Fig 7d). The HmAb64-bound gp120 subunits rotated outward comparing to those in b12- and ab1303-bound state (Fig. 7d). It is of note that the V1V2 apex was completely disordered (Fig. 7d, g). We did not observe densities for the gp120 bridging sheet between the V1V2 stem and β20/β21 (Fig. 7g), while it is present in other open form trimer structures ^33,35–37^, likely due to the binding mode of HmAb64, which approached gp120 with its heavy and light chains aligning along the Env 3-fold axis, In contrast, other open-occluded conformation-inducing antibodies, such as b12 and ab1303, bind to gp120 in modes that are almost perpendicular to the HmAb64 orientation with their heavy chain close to and light chain away from the 3-fold axis (Supplementary Fig. orientation). Most of the CDR loops of HmAb64 retain similar conformations as in the apo form except CDR H3, which has a shift of about 4.9 Å at the tip, and CDR L2 to fit its conformation into the pocket between the inner and outer domains of gp120 (Supplementary Fig 2). The overall epitope region of HmAb64 is extensive, with buried surface areas of 656 Å^2^ and 561 Å^2^, contributed by heavy and light chains, respectively (Fig. 7h). Among all the SHM residues, only two (Asn^L31^ and Thr^L51^) were involved in direct contact with gp120 (Fig. 7h). This region almost completely covers CD4bs and partially overlaps with the epitope regions of other CD4bs nAbs (Fig 7e), consistent with competition and binding analyses that HmAb64 is a CD4bs-specific Ab.

The cryo-EM structure also revealed details of HmAb64 antigen recognition that include a couple features that are considered signatural for CD4bs Abs as well as CD4 itself. First is the key recognition of the conserved Asp^368^ of gp120. CD4 itself and all members of the VRC01 family of bnAbs recognize Asp^368^ by forming a salt bridge ^14,17^ with a conserved Arg in the third framework region of the heavy chain. In the case of HmAb64, the salt bridge is formed between Asp^368^ and the CDR H3 residue Arg^H100e^ (Fig 7f). The second feature is the interaction with the Phe^43^ cavity region, next to Asp^368^, of gp120 (Fig 7e). CD4 plugs this cavity by the side chain of its Phe^43^ (hence the name of the cavity); some bnAbs, such as VRC-PG20, also insert the cavity with the side chain of a Trp residue. However, CD4bs bnAbs are highly variable in interacting with this region ^17^. For example, the archetype CD4bs bnAb VRC01 does not plug the cavity itself but engages the region with a loop of hydrophobic residues ^11^. In the case of HmAb64, it also approaches the region with a hydrophobic loop of CHRH3 centered at residue Leu^H100^ (Fig. 7f).

Unlike most CD4bs-specific nAbs that approach the trimer at angles favorable for the engagement of the closed form trimer, HmAb64 approaches its epitope using a distinct angle compatible with gp120 subunits rotating outward as in the open form conformation to minimize the steric hindrance from the neighboring gp120 ^17^. HmAb64 engaged gp120 primarily through its CDR H3 to contact with the pocket at the interface between inner and outer domains. In addition to the Phe^43^ cavity where CD4 binds, HmAb64 extends the hydrophobic interaction of its CDR H3 to the C-terminus of α1 helix (Fig. 7e), which is on the other side of the Phe^43^ cavity and is relatively inaccessible by Ab due to the arrangement of the β20/β21 loop in the closed form. Therefore, binding of HmAb64 causes a structural clash with the β20/β21 loop. This would explain why the gp120 bridging sheet was not observed in the cryo-EM reconstruction 3D map.

## Discussion

This is the first report that a CD4bs-specific mAb was isolated from a human volunteer who only received a course of experimental HIV vaccine within 8 months, and this human mAb has neutralizing activities against a panel of viruses from diverse subtypes. The cryo-EM structure revealed HmAb64 recognition to include features that are considered signatural for CD4bs Abs as well as CD4 itself. Human CD4bs-specific mAbs were also isolated in RV350 study from volunteers who received a late (6–8 years after the initial round of vaccination) boosting of RV144 vaccines with ALVAC-HIV and AIDSVAXgp120 B/E ^38^ but no CD4bs mAbs have been reported from the original RV144 study, or from other human HIV vaccine studies in literature.

Neutralizing antibody function has been considered for several decades as the most important protective mechanism for a prophylactic HIV vaccine ^12,39,40^. There are several well described regions on the envelope glycoprotein (Env) of HIV serving as the main targets of neutralizing antibodies ^12,40–43^. The CD4bs is considered the most critical as CD4bs specific mAbs isolated from HIV infected patients showed potent and broadly neutralizing activity ^4,16,17,44,45^. At the same time, the CD4bs is a conformational epitope formed by non-linear amnio acid sequences located at different regions of Env making it a difficult immunogen to be produced by the conventional subunit protein vaccine designs. Significant effort has been devoted to the design and testing of novel immunogens to elicit CD4bs antibodies, which have not been successful in eliciting neutralizing antibodies, especially in human vaccine volunteer studies. Therefore, the success of isolating a CD4bs mAb from a healthy volunteer who received an HIV vaccine is a milestone in the development of HIV vaccines.

This isolation of a CD4bs mAb from the PDPHV vaccine is not totally unexpected based on our previous studies. We first discovered that CD4bs antibodies were present in rabbit sera immunized by the DNA prime/protein boost but not protein alone HIV vaccine ^23^ based on the serum competition assay against known CD4bs mAb b12. That finding supported the long-held belief that DNA immunization can deliver antigens with better conformation comparing to the same antigen delivered in the form of a recombinant protein. A nucleic acid vaccine like DNA vaccine may be particular useful to preserve the conformation of CD4bs-related epitopes because the in vivo production and folding of Env protein and proper post-translational modifications such as glycosylation may better mimic the native Env structure than the recombinant Env protein produced and purified by the in vitro process.

Our previous study also reported that sera of the DNA prime-protein boost DP6-001 study volunteers can compete against known CD4bs mAbs using the same binding competition assay ^46^. Control human sera from volunteers of HVTN041 elicited by recombinant gp120 protein alone vaccine showed high level V3-type antibodies but no CD4bs antibody responses ^46^. Isolation of CD4bs mAb in the current report from one of these volunteers provided more definitive evidence confirming the elicitation of CD4bs antibodies in DP6-001 volunteers.

More significantly, the new CD4bs mAb was able to neutralize a panel of primary HIV isolates of diverse subtypes and the neutralizing activities were quite potent against some of them. The total percentage of breadth against the standard panel is still quite moderate comparing to other well-known CD4bs mAbs such as VRC01. Two recently reported trial results (HVTN704/HPTN085) showed that VRC01 did not prevent overall HIV-1 acquisition more effectively than placebo, and only analysis of VRC-01-sensitive HIV-1 isolates provided proof-of-concept that bnAb prophylaxis can be effective ^47^. Therefore, it is more important to elicit CD4bs antibody responses with cross-subtype neutralizing activity as effective vaccine. At the same time, it also raises the question as how to translate the breadth of neutralizing antibody from in vitro assay to real-world protection which warrants further research. The real protection potential of PDPHV can only be demonstrated in an efficacy study.

Our cryo-EM structure of the HmAb64 in complex with a SOSIP trimer has further revealed the structural basis of its function. The molecular detail of the complex not only shows that epitope of HmAb64 falls into the CD4bs supersite previously identified ^17^, but also the characteristics of antigen engagement of typical CD4bs Abs, including that of the salt bridge of formed with Asp^368^ and hydrophobic interaction with Phe^43^ region of gp120. The cryo-EM structure also provided a structural explanation that HmAb64 is yet a bnAb, i.e., its engagement of the HIV-1 Env trimer is more compatible with the open form conformation and its approach angle creating some hindrance with the formation of the bridging sheet. Interestingly, the low resolution 3D reconstruction based on some negative stain EM for the human CD4bs-specific mAbs isolated in RV350 study from volunteers who received a late (6–8 years) boosting of RV144 vaccines with ALVAC-HIV and AIDSVAXgp120 B/E suggested that those mAbs also bind to the open form of Env trimer ^38^. However, it remains to be seen if those human mAbs have the typical characteristics of antigen engagement for CD4bs mAbs.

In summary, we have demonstrated that authentic neutralizing CD4bs antibodies can be induced in human by vaccination in a relatively short time. Definition of the recognition of HmAb64 reveals it to bind to a conformation between prefusion close and occluded open forms of Env trimer, demonstrating that even when recognition of the CD4bs on gp120 does occur, clashes with regions surrounding the CD4bs preclude recognition of the prefusion-closed conformation of the Env trimer, the conformation recognized by most broadly neutralizing antibodies.

## Methods

### DP6-001 human sera and PBMCs

The serum and peripheral blood mononuclear cell (PBMC) samples from DP6-001 trial were collected according to institutional review board (IRB) approved protocol ^22^. The serum and PBMC samples involved in the current study were from Subject 009 who received the HIV-1 DNA and protein vaccination comprised of full-length 120 Env antigens from HIV-1 clade A (92UG037.8), clade B (Bal and 92US715.6), clade C (96ZM652 and clade E (93TH976.17). The details of vaccine formulation as well as clinical safety and immunogenicity results were previously reported ^22^. The serum samples used in this study were collected prior to immunization (Pre-immune), at 2 weeks after the 3^rd^ DNA immunization (Post DNA-3, Week 14), 2 weeks after the 1^st^ protein boost (Post Prot-1, Week 22) and 2 weeks after the 2^nd^ protein boost (Post Prot-2, Week 30). The PBMCs used for Env-specific mAb isolation were collected at 2 weeks after the 2^nd^ protein boost (Week 30).

### Memory B cell isolation and immortalization

The cryopreserved PBMCs from Subject 009 were thawed and cultured in RPMI 1640 with 15% Fetal Bovine Serum (FBS), 1% L-glutamine and 1% Penicillin-Streptomycin (P/S) at 37°C with 5% CO2 overnight. The B cells from overnighted culture PBMCs were first enriched using MACS Human B Cell Isolation Kit II (Milteny Biotec, San Diego, CA). Then, CD27+ memory B cells were isolated using human memory B cell isolation kit (Milteny Biotec). For EBV immortalization of B cells, the memory B cells isolated from Subject 009 were seeded at 5 cells/well in 96-well U-bottom microplates in 200µl of complete RPMI medium containing 2.5µg/ml CpG ODN2006, in the presence of EBV (30% supernatant of B95-8 cells) and irradiated allogeneic mononuclear cells (50,000 per well) ^48^. After 12 days of culture at 37°C with 5% CO2, 50µl of culture supernatants were harvested and screened for gp120 binding by ELISA. The EBV transformed cells from the gp120 binding positive wells were picked and expanded for clonal culture. The antibody from positive wells were further determined by ELISA with anti-Lamda and anti-Kappa secondary antibody, separately. The cells from gp120-specific clone #64 were harvested and preserved in RNALater (Qiagen, Redwood City, CA) for RNA extraction and Ig gene cloning.

### Full length immunoglobulin IgG heavy and Kappa chain gene isolation

RNA was extracted from clone #64 cells using RNeasy Mini Kit (Qiagen)and eluted in DNase/RNase-Free water (25µl/million cells) for RT-PCR. The RT-PCR reactions were performed using OneStep RT-PCR Kit (Qiagen). Briefly, for 25 μl reaction, the following reagents were added to each tube: 12.5 μl 2x Sensiscript RT rxn mix (Qiagen), 0.1μl (4 units) of RNase inhibitor, 5 μl of #64 RNA sample, 0.5 μl of 5’ primer(s) and 0.5μl of 3’ primer for heavy chain or kappa chain, and 6μl of RNA-free water.

For heavy chain RT-PCR, the 5’ primers were mixture 7 primers (VH1-Leader-A, B, C, D, E, F and G, 5 nM/primer, Supplementary Table 2). The heavy chain 3’ primer was a single universal primer: CH2-IgG (20 nM) (Supplementary 2 Table). The RT-PCR reaction condition was at 50°C for 30 mins; 94°C for 2 mins, followed by40 reaction cycles (94°C for 15 second, 55°C for 30 second and 68°C for 100 second). For Kappa chain RT-PCR: 5’ primers were a mixture of 2 primers (Vk1/2-Leader-A and B, 10 nM/primer, Supplementary Table 2). The Kappa chain 3’ primer was a single universal primer: CL2-kappa (Supplementary Table 2). The RT-PCR reaction condition was 50°C for 30 mins; 94°C for 2 mins, followed by40 reaction cycles (94°C for 15 second, 55°C for 30 second and 68°C for 75 second). The PCR products for IgG heavy chain and kappa chain were analyzed by agarose gel electrophoresis. The 1.5 kb heavy chain and 0.7 kb kappa chain fragments were extracted using QIAquick PCR Microcentrifuge Protocol.

### Construction of heavy chain expression plasmid

To construct the mAb64 heavy expression plasmid, PCR was conducted using #64 heavy chain RT-PCR product as template, VH1-LEADER-HindIII as 5’ primer and CH2-IgG-NheI as 3’ primer. In 50μl reaction, the following reagents were added: 25μl of 2X Kapa HiFi mix (KAPA Biosystem), 10μl of #64 heavy chain RT-PCR product, 1µl of 5’ primer (VH1 LEADER-HindIII, 20 nM) and 1μl of 3’ primer (CH2-IgG-NheI, 20 nM), and 13µl of RNA-free water. The PCR reaction condition was 94°C for 2 mins, followed by 30 reaction cycles (94°C for 15 second 63°C for 30 second and 68°C for 100 second. The 1.5 kb heavy chain fragment was extracted from agarose gel. The heavy chain was cloned into mammalian expression vector pJW4303 HindIII and NheI cloning site ^49^and confirmed by DNA sequencing.

### Construction of Kappa chain expression plasmid

To construct the mAb64 Kappa expression plasmid, PCR was conducted using clone #64 Kappa chain RT-PCR product as template, Vk1/2-Leader-HindIII as 5’ primer and CL2-kappa-BamHI as 3’ primer. In 50 μl reaction, the following reagents were added: 25μl of 2X Kapa HiFi mix (KAPA Biosystem), 10μl of #64 kappa chain RT-PCR product, 1µl of 5’ primer (Vk1/2-Leader-HindIII, 20 nM) and 1μl of 3’ primer (CL2-kappa-BamHI, 20 nM), and 13µl of RNA-free water. The PCR reaction condition was 94°C for 2 mins, followed by 30 reaction cycles (94°C for 15 second, 63°C for 30 second and 68°C for 75 second. The 0.7 kb Kappa chain fragment was extracted from agarose gel. The kappa chain was cloned into expression vector pJW4303 HindIII and BamHI cloning site ^49^ and confirmed by DNA sequencing.

### Expression and production of mAb64 mAb

To express and produce the mAb64, heavy and light chain plasmids (mAb64-HC/pJW4303 and mAb64-KC/pJW4303, 1:1 ratio) were transfected into Freestyle 293F cells (Invitrogen) as previously described ^50^. At 2 days after transfection, supernatant was harvested. The human IgG (HmAb64) antibody was purified with an AKTA FPLC using Protein A HP columns (GE Healthcare). The quality of purified HmAb64 IgG was analyzed by SDS-PAGE under reduced and non-reduced conditions.

### ELISA

ELISA was conducted to examine the HIV-1 Env-specific bindings in DP6-001 serum samples, EBV-transformed B cell media and the final mAb64 against gp120, gp120-Core or their D368R mutant protein as previously described ^22^. To detect the polyreactivity of monoclonal antibodies, ELISA plate was coated with ssDNA, dsDNA and LPS and Insulin with final concentration of 10μg/ml for ssDNA, dsDNA and LPS, and 5μg/ml for Insulin in PBS, 50 μl/well, overnight at RT. After blocking for 2 hours at RT, properly diluted HmAb64 (50μl/well) were Incubated for 2 hours at RT. HRP-conjugated goat anti-human IgG (Jackson, 109-035-098) in dilution buffer (1:1000, 50 μl/well) was incubated for 1 hour at RT. At last, plate was developed for 5 minutes at 37°C in 100 µl of a 3,3’5,5’-tetramethylbenzidine substrate solution (Sigma). The reaction was stopped with 50 µl of 2N H2SO4. Plates were read at 450 nm.

### Binding affinity analysis with Octet Qke

To evaluate the HmAb64 binding kinetics to different gp120 proteins, Octet Qke (ForteBio) assays were conducted based on biolayer interferometry. Briefly, the HmAb64 antibody was loaded on to Protein G (ProG) sensor tips at 20µg/mL in kinetics buffer. After capture, biosensor tips were washed in kinetics buffer and a baseline measurement recorded. The sensors were then incubated with serial diluted gp120 protein (600nM-9.3 nM) to measure the association rate (K_on_) and dissociation rate (K_off_). The antibody binding kinetics and KD values (K_off_/K_on_) were analyzed by the ForteBio Data Analysis software package v7.1 using a 1:1 binding model.

### Neutralization Assays

Two types of neutralization assays were performed to evaluate HmAb64 neutralizing activities. 1) TZM-bl cell based HIV-1 pseudovirus neutralization assays were performed using the previously validated protocol in TZM-bl cells ^51^ by the laboratories of Dr. David Montefiori at Duke University and Dr. Michael Seaman at Harvard Medical School. HIV-1 pseudoviruses were produced in HEK293T cells by co-transfection of a plasmid expressing gp160 envelope with the pSG3ΔEnv backbone vector. A panel of 23 pseudoviruses expressing HIV-1 Env were evaluated (Table 1). Serial dilutions of HmAb64 were performed in duplicate, followed by addition of pseudovirus. Plates were incubated for 1 hour at 37°C followed by addition of TZM.bl target cells (1×10^4^/well) and DEAE dextran (final concentration 11 μg/ml). Plates were incubated at 37°C for 48 hours then developed with Promega Bright-Glo luciferase assay reagent per the manufacturer’s instructions. Relative Light Units (RLUs) were collected by running plates on a Promega GloMax luminometer. Neutralization was calculated as a percent reduction in luciferase activity in the presence of antibody compared to the luciferase activity induced by the virus control without antibody, calculated as following: [(1-(Average sample RLUs-Cell Control RLUs)/(Virus Control RLUs-Cell Control RLUs)]x100. 2) For the large standard panel of 208-Env pseudoviruses, neutralization assays were performed on a Beckman Coulter Biomek automated liquid handler integrated with Thermo Cytomat Incubator (37°C and 5% CO_2_) and Molecular Devices Spectramax Paradigm luminometer at the Vaccine Research Center, NIH as described previously ^52^. The automated assay workflow miniaturized the previously optimized HIV-1 neutralization assay system in the TZM-bl cells ^51^ in a 384-well plate format. The automated 384-well assay performed sample addition and dilution, virus addition with 45 minutes incubation, followed by TZM-bl cell addition and 48-hour incubation, and the subsequent supernatant removal and addition of luciferase substrate followed by luminescence measurement.

### Virus capture competition

The ability of human immune sera to inhibit the capture of pseudovirions by the CD4bs specific mAb b12 was investigated using a previously described competition assay ^23^. Pseudotyped virions in this assay expressing both HIV-1 Env and vesicular stomatitis virus G protein on the virion surface were produced. The vesicular stomatitis virus G protein will mediate the entry of virions into the target cell line CF2.CD4.CCR5 irrespective of any neutralizing activity against the HIV envelope present in the sera, thus providing a sensitive readout of captured virus as previously described (24). The capture of pseudovirions by b12 will be blocked if there is a competing CD4bs antibody in the human sera. Specifically, microwells were coated with b12 (5 μg/ml) overnight and were then washed and blocked with 3% bovine serum albumin in PBS. Graded dilutions of human immune sera were added to the virus, and the virus-serum mixtures were then added to b12-coated ELISA wells for 3 h, followed by washing with PBS. CF2.CD4.CCR5 cells were overlaid, and 2 days later, infection was measured by assaying luciferase. The reciprocal serum dilution that inhibited b12-mediated virus capture by 50% was recorded.

### mAb binding to HIV infected CD4^+^ T cells

Primary CD4^+^ T cells were isolated from PBMCs of healthy donors by negative selection (StemCell). Cells were cultured in 5 days in media containing anti-CD3, retinoic acid and IL2 (20u/ml). On day 5, cultures were with 4µl of HIV SF162 per 10^6^ cells (stock concentration 160ng/ml). After 3 days, cells were stained with mAbs A32 (1.5µg), VRC01 (2µg) (positive controls), human IgG1 (negative control), and HmAb64 (2µg), washed and incubated with 1µl of goat anti-human -PE (Southern Bio) and data collected on a FACS CANTO (BD Biosciences) using standard protocols.

### HmAb64 blocking of gp120 binding to CD4^+^ T cells

The gp120s derived from HIV-1 MW959, SF162, Bal and SIVmac251 were biotinylated (Lynx biotinylation kit, Biorad) and used to stain primary CD4^+^ T cells, isolated from healthy donors, followed by neutravidin PE (Invitrogen) in the absence or presence of the CD4 mAb Leu3A (positive control) or HmAb64, washed 2x and data was collected on a FACS CANTO using standard protocols.

### Surface plasmon resonance analysis of CD4 or anti-Env mAb binding to surface immobilized BG505-SOSIP, Bal gp120 or A244 gp120

Experiments were performed using a Biacore 3000 instrument (Cytiva Life Sciences) using CM4 or CM5 sensor chips. The data were evaluated using BIAevaluation 4.1.1 software (Cytiva Life Sciences). The chip surface was activated by injecting 35 μl of a 1:1 mixture of 0.05 M *N*-hydroxysuccinimide and 0.2 M *N*-ethyl-*N*-(dimethylaminopropyl) carbodiimide at 5 μl/min. HIV gp120 or BG505-SOSIP at concentrations of 5 μg/ml in 10mM NaOAc, pH 4.5, were immobilized to approximately 750 resonance units (RU). After the proteins were immobilized to the desired densities, unreacted sites on each surface were blocked with 35 μl of 1 M Tris-HCl (pH 8.0). One surface was activated and blocked without ligand to act as a control surface for non-specific binding of the soluble ligand. Any binding was subsequently subtracted from the remaining surfaces. Running buffer was HBS (pH 7.4), 3mM EDTA, 0.005% Tween- 20, 0.05% soluble carboxymethyl-dextran. Binding experiments were carried out at a flow rate of 25 μl/min at 25 °C. After a 2 min injection, the surface was washed for an additional 2 min in running buffer to follow dissociation of the bound ligand from the surface. The surfaces were regenerated by multiple injections of 4.5 M MgCl_2_ at a flow rate of 100 μl/min.

### Inhibition of anti-gp120 mAb binding to HIV-1 Envs by pre-bound sCD4 as measured by surface plasmon resonance analysis

Inhibition of anti-gp120 mAb binding to HIV-Env by sCD4 was performed by passing either buffer or sCD4 (D1D2) at the indicated concentrations over the surfaces described above at a flow rate of 25 μl/min at 25 °C, followed by a second sequential injection of a single concentration of the indicated mAb. The ability of sCD4 to inhibit the anti-Env mAb was determined by comparing the binding of the antibody in the absence or presence of the pre-bound sCD4 as described ^53^.

### Fab production and purification

The Fab fragment of HmAb64 was prepared by papain digestion as described previously ^54^ for crystal structure analysis. Briefly, the IgG molecule was mixed with papain (Worthington) at a 20:1 ratio and 100 mM Tris (pH 6.8) with 1 mM cysteine hydrochloride and 4 mM EDTA. The mixture was incubated for 1 hr at 37°C and the reaction was stopped by adding 10 mM iodoacetamide. The Fab fragment was separated from Fc and the undigested IgG by a protein A column and further purified by size exclusion chromatography. The concentration of the Fab fragment for crystallization was 10 mg/mL.

### Crystallization, data collection, structure determination and refinement

The preliminary crystals of HmAb64 Fab alone were obtained by robot screening using the vapor diffusion hanging drop method as previous described ^55,56^. Crystals of HmAb64 Fab alone were obtained in a well solution of 14% polyethylene glycol 8000, 0.1 M HEPES pH 7.5, and 8% ethylene glycol. Crystals of HmAb64 Fab were first soaked in the mother liquor with additional 20% glycerol (v/v) and 20% ethylene glycol, respectively, before placed in the X-ray beam. Data of the crystals of HmAb64 Fab alone were collected at the synchrotron beamline X6A at the National Synchrotron Light Source (NSLS), Brookhaven National Laboratory. All data sets were processed using the HKL 2000 software package ^57^, and structures were determined by molecular replacement as we previously determined (PDB ID 4JO1) ^55,58^. Cycles of refinement for each model were carried out in COOT and Phenix ^59,60^. Figures were generated using PyMOL ^61^.

### Expression and production of scFv HmAb64

To aid cryo-EM studies of the HmAb64-Env complex while preventing aggregation caused by the antibody constant region, a Single-chain Fv (scFv) version of HmAb64 was generated. This involved linking the VH and VL domains using 3x G4S linkers and adding a His tag to the C-terminus. The resulting construct was cloned into the pVRC8400 vector. After expression in Expi293 cells, the protein was purified from the conditioned media using cOmplete His-Tag Purification Resin (Roche) via affinity purification. Further purification was then performed using size exclusion chromatography (SEC).

### Design and purification of CNE40 Env trimer and production of a scFv-HmAb64 complex

Single-chain Fc (scFc) tagged CNE40 Env trimer was generated by fusing the N-terminus of gp120 to knob-in-hole scFc domain, separated by an HRV3C site. The protein was expressed in Fresstyle293 cells, at 37°C for 5 days. The complex of scFv-HmAb64 and CNE40 Env trimer complex was produced by on-column HRV3C digestion. scFc-CNE40 supernatant was loaded on a protein A column and washed with PBS before adding purified scFv-HmAb64. Overnight incubation with HRV3C protease at 4°C was performed to release the complex from the resin. The flowthrough was concentrated and further purified by size exclusion chromatography (SEC) on a Superdex 200 Increase 10/300 GL column (GE Healthcare, Boston, MA) equilibrated in PBS. Complex formation was verified by SEC and SDS-PAGE in non-reducing and reducing conditions.

### Cryo-EM sample preparation, data collection and processing

A 3 μL of the scFv-HmAb64-CNE40 SOSIP protein complex, at 2.0 mg mL^−1^, was applied onto a C-Flat 1.2/1.3 copper grid (Electron Microscopy Sciences, EMS), which had been freshly glow-discharged at 15 mA for 25 s using a PELCO easiGLOW system (TED PELLA). The sample grid was blotted with filter paper (Whatman) for 1.5 s at 4°C under 100% relative humidity before plunging into liquid ethane using Vitrobot™ Mark IV (FEI).

Micrographs were collected on a Titan Krios (FEI) operated at 300 kV with a K3 direct electron detection camera (Gatan). An energy filter with a 20 eV slit width was used through imaging. Leginon ^62^ was used for ice thickness targeting and automated data collection. Each micrograph was collected at 105,000x nominal magnification resulting in a calibrated pixel size of 0.426 Å, using a dose rate of 35.47 e^−^·Å^−2^·s^−1^ with a total exposure of 1.6 s, for an accumulated dose of 56.76 e^−^·Å^−2^ over 40 frames. Due to a strong preferred orientation of the particles, a total of 16.962 micrographs were collected from 0°, 30°, 40° and 45° tilted angles, at a nominal defocus range of −5.0 to 8.1 μm.

Micrograph frames were aligned and dose weighted using MotionCor2 ^63^, and initial contrast-transfer-function (CTF) for each micrograph was estimated with CTFFIND4 ^64^in the Appion ^65^ platform for real-time data evaluation and pre-processing. Particle picking was trained in Warp ^66^. Further processing including 2D classification, 3D refinement and map sharpening was performed using cryoSPARC v 3.3.0 ^67^. Briefly, a total of 1,859,663 Warp extracted particles were sorted through 2 rounds of reference-free 2D classification to remove junk containing false picks and denatured/dissociated particles (Supplementary Figure 3). A total of 655,074 selected particles from the classes that exhibited Fv-bound trimer features were further sorted into four classes by heterogeneous refinement using an Ab-initio model low-pass filtered to 20 Å without importing symmetry (C1). The 259,148 particles belonging to the best class, which showed well-defined densities at the interface between gp120 and HmAb64 were subjected to 3D non-uniform (NU) refinement. Base on visual inspection, the reconstruction map exhibited a 3-fold symmetry. Therefore, the same set of 655,074 particles was re-processed with C3 symmetry imposed. Multi-class Ab-initio reconstruction (4 classes) was initially performed, followed by heterogeneous refinement (2 classes). The class of 316,221 particles was selected to conduct NU refinement with per-particle defocus and per-group CTF optimization. The resulting map reached an average resolution of 3.74 Å, based on the gold-standard Fourier shell curve (FSC) using a correlation cut-off of 0.143 ^68^.

### Model building and refinement

To create an initial starting model for CNE40 SOSIP, the gp120 and gp41 subunits of a homologous SOSIP (PDB ID 6CK9 ^69^; chain D and A, respectively) were used as structural templates and replaced with the corresponding sequences in SWISS-MODEL^70^. Due to poor resolution at the regions corresponding to the trimer apex in the final 3D reconstruction map, the V1V2, V3, and C4 (β20-β21) domains of the initial gp120 model were truncated before being fitted into the map using UCSF Chimera v1.13.1 ^71^. This model, without scFv, was first refined using Phenix v1.20.1 ^72^ by a single round of rigid body refinement, morphing, and simulating annealing, followed by rounds of manual inspection and model building in COOT v0.8.9.2 ^73^. The portion of HmAb64 scFv was then modeled using the crystal structure (Fv domains of PDB ID 6W73 ^74^), by fitting V_H_ and V_L_ domains separately into the map in Chimera, followed by manually adjustment, particularly, of the loops at the interface regions. After multiple iterations of model building and real space refinement in COOT and Phenix, respectively, the glycan resuides were added based on interpreted map densities using Glyco module in COOT ^75^. The final refinement was carried out by real space and B-factor refinement with secondary structure restraints using Phenix. Model validation was performed using MolProbity^76^. The Cα RMSD was calculated using the script available in the PyMOL wiki. All the figures were created using UCSF Chimera X ^77^ or PyMOL v.2.3.3 (The PyMOL Molecular Graphics System, Version 2.3 Schrödinger, LLC.).

## Supplementary Material

Supplement 1
