# Supplementary material for: Human CD4-Binding Site Antibody Elicited by Polyvalent DNA Prime-Protein Boost Vaccine Neutralizes Cross-Clade Tier-2-HIV Strains
Source: Res Sq. 2023 Oct 20:rs.3.rs-3360161. Preprint. [Version 1] doi: 10.21203/rs.3.rs-3360161/v1 (PMC10602183; doi:10.21203/rs.3.rs-3360161/v1)
Supplement: Supplement 1 [file NIHPPrs3360161v1-supplement-1.pdf]

Supplementary Files

This is a list of supplementary files associated with this preprint. Click to download.

- mAb64tableonly20230912.pdf
- mAb64supplfiguresandtables20230912.pdf
